# Clinical Features of Actinomycosis in Older Adults: A 10-Year Experience of a Single Institute in Beijing, China

**DOI:** 10.7759/cureus.65734

**Published:** 2024-07-30

**Authors:** Ning Zhang, Kanghao Zhou, Xiu Gao, Xinmin Duan, Shengmin Yang, Lin Kang

**Affiliations:** 1 Department of Geriatrics, Peking Union Medical College Hospital, Peking Union Medical College, Chinese Academy of Medical Sciences, Beijing, CHN; 2 Department of Internal Medicine, Peking Union Medical College Hospital, Peking Union Medical College, Chinese Academy of Medical Sciences, Beijing, CHN; 3 Department of General Medicine, The First Hospital of Yulin City, Yulin, CHN; 4 Department of Infectious Diseases, Peking Union Medical College Hospital, Peking Union Medical College, Chinese Academy of Medical Sciences, Beijing, CHN; 5 Department of Endocrinology, Peking Union Medical College Hospital, Peking Union Medical College, Chinese Academy of Medical Sciences, Beijing, CHN

**Keywords:** treatment choices, difficult diagnosis, clinical characteristics, older adult, multiorgan actinomycosis

## Abstract

Background:Actinomycosis is a rare infectious disease with non-specific clinical presentations often resulting in delayed diagnosis, especially in older adults. Diagnosing and treating actinomycetal infections in this population can be particularly challenging due to the lack of comprehensive case series studies focusing specifically on actinomycosis in older adults. The existing literature mainly consists of case reports, highlighting the need for more extensive research in this area. This study aimed to provide a profile of actinomycosis in older adults to guide future research efforts.

Methods: Elderly patients aged 60 years and older who satisfied the inclusion criteria for actinomycosis at Peking Union Medical College Hospital from January 2014 to May 2024 underwent a retrospective analysis. The research centered on describing the clinical features and diagnostic techniques, distinguishing between different conditions, and treating clinically important instances of actinomycosis within this specific age bracket.

Results: This study involved 22 patients, with a balanced gender distribution of 11 males and 11 females, aged between 60 and 84 years, and a median age of 67 years. The disease predominantly affected the thoracic region (n=17), followed by the abdominal-pelvic (n=2) and orocervicofacial (n=2) regions, along with one case involving soft tissue (n=1). Microbiological methods confirmed the diagnosis in 17 cases (77%), while histopathological examination was employed in the remaining five cases (23%). General symptoms, such as fever and weight loss, were reported by 64% of the patients, whereas 32% exhibited symptoms localized to the infection site. Only one patient (4%) did not present any symptoms. The median duration from the onset of initial symptoms to diagnosis was 120 days (IQR 34.5-240). Nine patients were successfully treated with antibiotics, with only one patient experiencing a relapse during the follow-up period.

Conclusions: Infections caused by actinomycetes are infrequent among the elderly and often exhibit non-specific clinical symptoms and imaging results. Among the various types of actinomycetal infections in this demographic, pulmonary actinomycosis is the most prevalent. Recognizing the wide-ranging capacity of actinomycetes to induce infections beyond our present knowledge is essential. It is important for healthcare practitioners to deepen their knowledge of actinomycosis to prevent delays in both diagnosis and treatment.

## Introduction

Actinomycosis is a rare bacterial disease that has been known for over a century. The filamentous gram-positive bacilli, *Actinomyces* spp., are typically found in the human oropharynx, gastrointestinal tract, and urogenital tract. Actinomycosis can manifest in various clinical presentations, affecting different anatomical sites such as the face, bone and joint, respiratory tract, genitourinary tract, digestive tract, central nervous system, skin, and soft tissue structures [1]. The bacteriological identification of *Actinomyces* from a sterile site confirms the diagnosis of actinomycosis. However, isolating and identifying these causative bacteria only happens in a minority of cases. The high failure rate of culture can be attributed to factors such as previous antibiotic therapy, inhibition of *Actinomyces* growth by other microorganisms, suboptimal culture conditions, or insufficient short-term incubation [2]. Gram staining and the pathology of infected tissue are crucial for diagnosing actinomycosis. *Actinomyces* spp. invade tissues, leading to a chronic granulomatous infection characterized by the formation of small clumps known as sulfur granules due to their yellow color. Histopathological examination reveals basophilic masses with eosinophilic terminal clubs when stained with hematoxylin and eosin. Microscopic findings typically show necrosis with yellow sulfur granules and filamentous gram-positive fungal-like pathogens. These yellow sulfur granules are composed of a conglomeration of bacteria trapped in biofilm [3].

The elderly population is characterized by a wide range of health conditions, including multiple chronic illnesses and geriatric syndromes like frailty and sarcopenia. Diagnosing and treating actinomycetal infections in this demographic presents unique challenges. Existing literature primarily consists of case reports [4-15], with a notable absence of case series studies. To address this gap, a retrospective analysis was conducted on cases of actinomycete infection among individuals aged 60 and above at Peking Union Medical College Hospital from 2014 to 2024. This study examined infection sites, diagnostic methods, comorbidities, treatments, and outcomes associated with actinomycete infections in the elderly. The findings aim to enhance clinicians' awareness and knowledge of this particular type of infection in older adults.

## Materials and methods

The study was reviewed and approved by the Ethics Committee of Peking Union Medical College Hospital (approval number: I-23PJ738). Hospitalized patients aged over 60 years with a discharge diagnosis of "actinomycosis" were identified from the Hospital Information System database of Peking Union Medical College Hospital between January 1, 2014, and May 30, 2024. Diagnostic criteria for actinomycosis included (1) detection of actinomycetes in pathological tissue specimens obtained from surgical excision, bronchoscopic biopsy, or bronchoalveolar lavage; (2) isolation of actinomycetes from sterile tissues such as pleural, abdominal, or pleural drainage fluids; and (3) isolation of actinomycetes from lower respiratory tract sputum or bronchoalveolar lavage cultures, accompanied by clinical improvement with anti-actinomycete therapy. The diagnosis of all cases was confirmed by a multidisciplinary team composed of experts from the departments of infectious disease, respiratory medicine, microbiology, and radiology.

**Figure 1:**
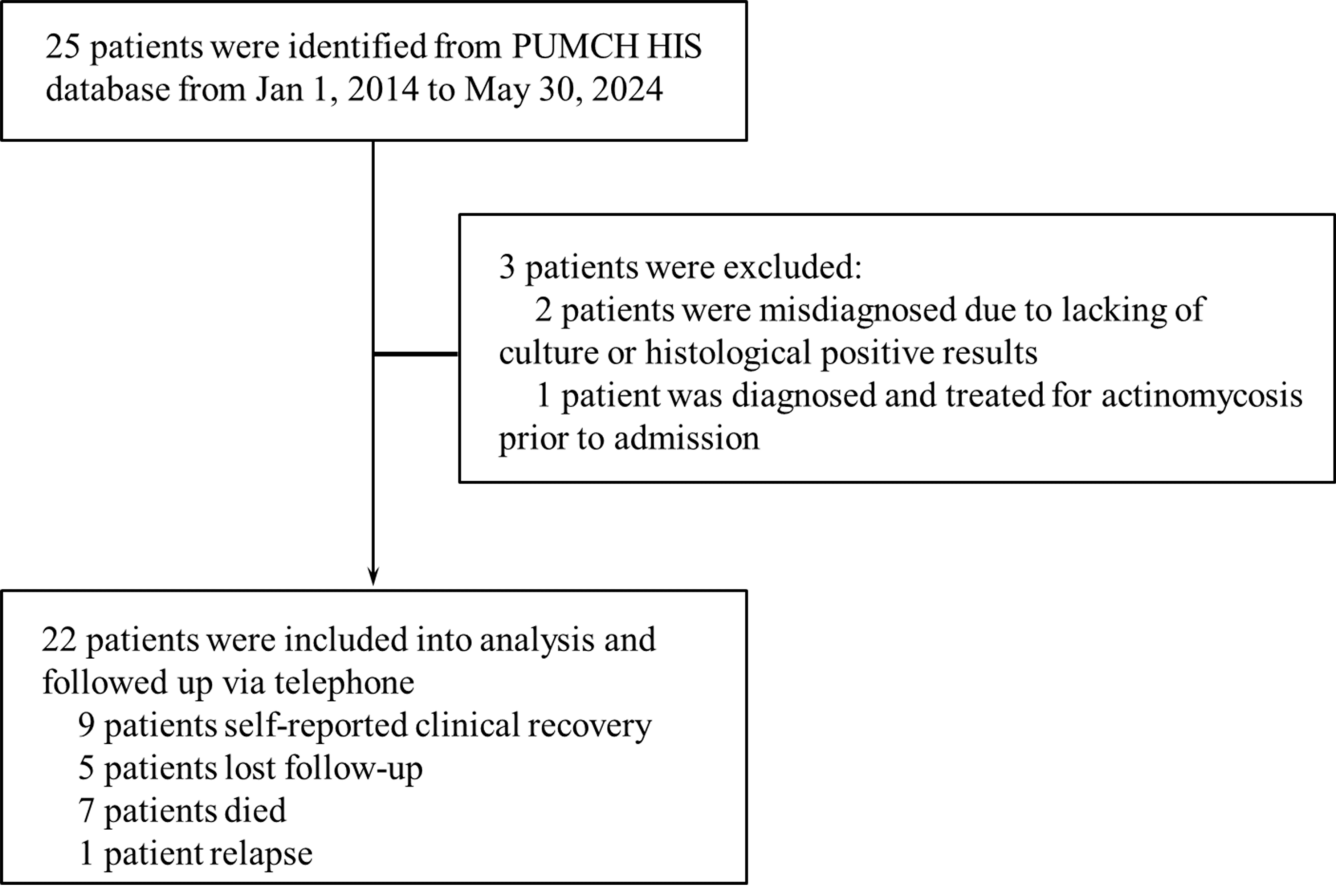
Screening and enrollment of patients PUMCH HIS: Peking Union Medical College Hospital Hospital Information System

Exclusion criteria for this study included (1) histological or culture-positive results without typical clinical manifestations or imaging features of actinomycosis and (2) patients who were already diagnosed with actinomycosis prior to hospitalization or those with a history of actinomycosis that had been successfully treated. Data for each patient were retrospectively collected, including gender, age, medical records review, clinical manifestations, infection foci, local or systemic risk factors for infection, diagnostic criteria, antibiotic regimens, and clinical responses. Furthermore, the study also documented the presence of multiple comorbidities in these patients. The age-adjusted Charlson comorbidity index (aCCI) was applied to evaluate the severity of comorbidities, where higher scores indicated a higher level of complexity [16]. Subsequently, telephone follow-ups were performed with all patients in May 2024. Complete resolution of clinical symptoms was defined as a successful outcome, while causes of death for deceased patients were examined and documented (Figure 1).

## Results

After searching the medical big data platform of Peking Union Medical College Hospital, a total of 25 medical records of actinomycete infections in individuals aged 60 and above were identified. Following multidisciplinary consultation, a total of 22 patients were included in the study (Figure 1). The gender distribution was balanced, comprising 11 male and 11 female patients. The median time from the onset of clinical symptoms to diagnosis was 120 days, with an interquartile range of 35 to 240 days. Additionally, the median aCCI was 4 points. Table 1 and Table 2 outline the clinical characteristics of 22 elderly patients diagnosed with actinomycete infection. Among them, eight patients exhibited local risk factors such as poor oral hygiene, surgery, local trauma, and intrauterine device (IUD) insertion. Common risk factors observed in 14 patients included a long-term smoking history in eight patients, type 2 diabetes in four patients, glucocorticoids combined with immunosuppressants in three patients, alcohol consumption in three patients, glucocorticoid therapy in one patient, immunosuppressant therapy in one patient, malignant tumors in two patients, and tuberculous lymphadenitis in one patient. It is worth noting that all patients tested negative for HIV infection.

**Table 1:** Summary of 22 cases of actinomycosis WBC: white blood cell, ESR: erythrocyte sedimentation rate, hsCRP: high-sensitivity C-reactive protein, F: female, M: male, NR: not reported

No.	Sex	Age, years	Presentation	Main symptoms	WBC count, × 10^9^/L	hsCRP value, mg/L	ESR mm/h	Diagnoses initially suspected	Diagnostic time frame, days	Diagnosis
Case 1	F	70	Thoracic	Fever+local symptoms	14.2	108.5	85	Community-acquired pneumonia	62	Sputum culture+chest imaging
Case 2	M	76	Thoracic	Fever+local symptoms	4.4	0.9	13	Community-acquired pneumonia	22	Sputum culture+chest imaging
Case 3	M	60	Thoracic	Fever+local symptoms	10.2	0.6	69	Community-acquired pneumonia	25	Sputum culture+chest imaging
Case 4	F	67	Thoracic	Local symptoms	6.1	11.8	54	Community-acquired pneumonia	67	Sputum culture+chest imaging
Case 5	M	64	Thoracic	Fever+local symptoms	8.5	53.1	91	Pulmonary space-occupying lesion	270	Bronchoalveolar lavage fluid culture
Case 6	M	61	Thoracic	Local symptoms	4.7	1.2	6	Pulmonary space-occupying lesion	42	Sputum culture
Case 7	F	60	Thoracic	Fever+local symptoms	6.5	1.2	19	Community-acquired pneumonia	14	Bronchoalveolar lavage fluid culture
Case 8	M	63	Thoracic	Fever+local symptoms	10.2	43.7	96	Lung abscess	180	Histology
Case 9	F	62	Thoracic	Local symptoms	5	4.5	20	Pulmonary nodule	270	Bronchoalveolar lavage fluid culture
Case 10	F	71	Thoracic	Fever	1.3	0.6	28	Lung abscess	30	Sputum culture
Case 11	F	66	Thoracic	None	4.9	0.3	9	Pulmonary space-occupying lesion	730	Bronchoalveolar lavage fluid culture
Case 12	F	67	Thoracic	Local symptoms	7	3.2	60	Asbestosis	153	Bronchoalveolar lavage fluid culture+sputum culture
Case 13	M	72	Thoracic	Fever+local symptoms	6.8	1.4	17	Relapsing polychondritis	180	Bronchoalveolar lavage fluid culture
Case 14	F	81	Thoracic	Fever+local symptoms	5.7	11	10	Community-acquired pneumonia	51	Sputum culture+chest Imaging
Case 15	M	67	Thoracic	Fever+local symptoms	12.8	31.1	NR	Pneumonia	30	Bronchoalveolar lavage fluid culture
Case 16	M	67	Thoracic	Local symptoms	25.4	218.1	66	Tuberculosis	219	Pleural fluid culture
Case 17	M	68	Thoracic	Fever+local symptoms	8.5	24.6	NR	Lung cancer	286	Histology
Case 18	F	74	Abdominopelvic	Local symptoms	11.5	NR	27	Cervical intraepithelial neoplasia	36	Histology
Case 19	M	67	Abdominopelvic	Fever	17.6	151.2	83	Abdominal aortic stent infection	150	Blood culture
Case 20	M	63	Orocervicofacial	Local symptoms	9.4	NR	NR	Pharyngeal tumor	240	Histology
Case 21	F	63	Soft tissue	Fever+local symptoms	6	64	90	Right foot abscess	90	Pathogenic culture of the debrided tissue
Case 22	F	84	Orocervicofacial	Poor appetite, weight loss	5.4	NR	NR	Granulomatous inflammation	240	Histology

**Table 2:** Summary of 22 cases of actinomycosis NR: not reported, ANCA: antineutrophilic cytoplasmic antibody, N/A: not applicable, IUD: intrauterine device

No.	Surgery	Antibiotics	Outcomes	Comorbid diseases	Local risk factors	Common risk factors	aCCI
Case 1	No	Tazocin+Imipenem	Died	Interstitial lung disease	NA/-	Glucocorticoids+immunosuppressants	3
Case 2	No	Levofloxacin+Sulfonamide	Cured	Hypertension, hyperlipidemia, cerebral infarction	NA/-	Smoking	5
Case 3	No	Piperacillin sodium-sulbactam sodium→Amoxicillin	Cured	Diabetes	NA/-	Diabetes+smoking	3
Case 4	No	Clindamycin+Levofloxacin	NR	Pulmonary interstitial fibrosis	NA/-	Glucocorticoids	4
Case 5	No	Imipenem→Amoxicillin+Moxifloxacin	Cured	ANCA-associated vasculitis, hypertension, reflux esophagitis, carotid artery stenosis, benign prostatic hyperplasia	NA/-	Glucocorticoids+immunosuppressants	3
Case 6	No	Amoxicillin＋Moxifloxacin＋Clarithromycin	Cured	Hypertension, reflux esophagitis, chronic bronchitis, cerebral infarction	NA/-	Smoking+alcohol consumption	5
Case 7	No	Sulfonamide→Clarithromycin＋Moxifloxacin＋Amikacin	Cured	Chronic bronchitis	NA/-	NA/-	3
Case 8	No	Penicillin→Erythromycin	Cured	Coronary heart disease, diabetes	Poor oral hygiene	NA/-	4
Case 9	No	Amoxicillin	Relapse	Atrial fibrillation, reflux esophagitis	NA/-	NA/-	3
Case 10	No	Amoxicillin	Died	Tuberculous lymphadenitis, diabetes, cholecystitis	Poor oral hygiene	Tuberculous lymphadenitis+diabetes	5
Case 11	No	Moxifloxacin+Doxycycline	NR	Hypertension, coronary heart disease, diabetes, sarcoidosis	NA/-	Diabetes, glucocorticoids+immunosuppressants	4
Case 12	No	Moxifloxacin→Amoxicillin	Died	Hyperlipemia	Poor oral hygiene	NA/-	3
Case 13	No	Amoxicillin	Died	Chronic obstructive pulmonary disease, relapsing polychondritis	NA/-	Smoking	4
Case 14	No	Tigecycline→Sulfonamide	Died	Rheumatoid arthritis, Sjogren's syndrome, liver cirrhosis	NA/-	Immunosuppressants	5
Case 15	No	Piperacillin sodium-sulbactam sodium→Amoxicillin	Died	Lung squamous carcinoma, hypertension, diabetes, coronary heart disease	NA/-	Lung squamous carcinoma+diabetes+smoking+alcohol consumption	7
Case 16	No	Amoxicillin clavulanate potassium→Amoxicillin	NR	Glaucoma, cataract, benign prostatic hyperplasia	NA/-	Smoking	4
Case 17	No	Amoxicillin clavulanate potassium→Amoxicillin	Cured	Gastric adenocarcinoma, hypertension lumbar disc herniation	Poor oral hygiene	Gastric adenocarcinoma+smoking+alcohol consumption	9
Case 18	Yes	Penicillin	Cured	Hypertension	IUD	NA/-	4
Case 19	No	Piperacillin sodium-sulbactam sodium→Penicillin＋Clindamycin→Penicillin+Imipenem+Caspofungin	Died	Coronary heart disease, abdominal aortic aneurysm	Poor oral hygiene+abdominal aortic stent infection	Smoking	4
Case 20	Yes	Levofloxacin+Sulfonamide	NR	Hypertension	NA/-	NA/-	3
Case 21	Yes	Amoxicillin	Cured	Rheumatoid arthritis, dilated cardiomyopathy	Local trauma	NA/-	3
Case 22	Yes	Tinidazole	NR	Osteoporosis	Local trauma	NA/-	3

Among the 22 elderly patients diagnosed with actinomycete infection, 14 experienced general symptoms, with 11 of them presenting fever during the course of the disease. One patient reported a loss of appetite and weight loss. Seven patients exhibited only local symptoms at the injection site. Notably, one patient remained asymptomatic but was discovered to have space-occupying lesions in the lungs during a physical examination, leading to a diagnosis of pulmonary actinomycosis. Elevated white blood cell counts were observed in seven patients, while 11 patients had elevated high-sensitivity C-reactive protein levels and 11 had elevated ESR levels. The median time from the onset of initial symptoms to diagnosis was 120 days (IQR 34.5-240).

In terms of actinomycete infection sites, the lungs were the most commonly affected. Out of 22 patients, 17 (77.3%) had pulmonary actinomycosis, all presenting with respiratory symptoms. The most prevalent clinical symptom was cough, followed by sputum production and hemoptysis. Cases 10 and 12 initially exhibited fever, cough, and yellow sputum, with both individuals also dealing with type 2 diabetes and poor oral hygiene. Chest CT scans revealed thick-walled cavities and patchy shadows in the lungs, leading to an initial diagnosis of lung abscess in both cases. Case 11 showed a nodule in the upper lobe of the right lung, initially suspected to be a lung tumor. However, further investigation through bronchoscopy ruled out the tumor, and actinomycete infection was confirmed through bronchoalveolar lavage fluid culture. Cases 1, 2, 3, 4, 7, and 14 were initially diagnosed with community-acquired pneumonia. Among them, cases 1, 2, 3, 4, and 14 were later diagnosed with pulmonary actinomycosis via sputum culture, while case 7 was diagnosed through bronchoalveolar lavage fluid culture. Case 5 presented with antineutrophil cytoplasmic antibody (ANCA)-associated vasculitis and interstitial lung disease, treated with glucocorticoids and immunosuppressants. Subsequent follow-up revealed new lung lesions, leading to the confirmation of pulmonary actinomycosis through culture of bronchoalveolar lavage fluid. Cases 6 and 9 presented with hemoptysis, with pulmonary actinomycosis confirmed through sputum culture and bronchoalveolar lavage fluid culture, respectively. Case 12, initially diagnosed with interstitial pulmonary lesions due to asbestosis, also had pulmonary actinomycosis confirmed through sputum culture and bronchoalveolar lavage fluid culture. Case 15 was diagnosed with actinomycosis through bronchoalveolar lavage fluid culture and later diagnosed with lung squamous cell carcinoma through a bronchoscopic mucosal biopsy. In case 16, chest CT revealed empyema, and actinomycetes were identified in the bacterial culture of pleural effusion, leading to a diagnosis of pulmonary actinomycosis. Case 17, a 68-year-old male, showed multiple soft tissue density shadows in the left lung apex segments on a contrast-enhanced chest CT. Biopsies revealed sulfur particles with irregular edges, confirming pulmonary actinomycosis. Notably, this patient also had gastric adenocarcinoma and received tumor-directed therapy alongside antiactinomycetal therapy.

Among the 22 elderly patients with actinomycete infection, two patients presented with abdominal and pelvic infections. Case 18, who had an IUD inserted, was diagnosed with a cervical actinomycete infection through a hysteroscopic biopsy. The infection was successfully treated with penicillin. Case 19 was hospitalized due to a fever and had previously undergone abdominal aortic stent placement for an abdominal aortic aneurysm a year prior. The patient had poor oral hygiene and a long history of heavy smoking. Following admission, enhanced CT imaging of the abdomen and pelvis revealed inflammatory changes in the abdominal aorta wall. Additionally, both peripheral blood cultures tested positive for actinomycetes. The final diagnosis was infective aortitis resulting from an actinomycete infection of the artificial abdominal aortic stent.

Case 21 had a history of receiving pedicures and developed necrotizing myofasciitis in the right foot three months later. The patient then underwent surgical debridement, and the pathogenic culture of the debrided tissue confirmed an actinomycete infection.

Regarding head and neck infections, case 20 presented with a pharyngeal mass and underwent surgical removal under general anesthesia. The postoperative pathology confirmed an actinomycete infection. Case 22 involves an 84-year-old woman who previously received zoledronic acid treatment for osteoporosis, followed by a right mandibular resection due to bisphosphonate-related osteonecrosis of the jaw. Subsequently, she developed soft tissue hyperplasia on the right cheek, leading to surgical resection. The postoperative pathological diagnosis revealed an *Actinomyces* infection.

Actinomycetes infection was not initially suspected in any case at the time of diagnosis. The most common initial diagnoses included infectious diseases, particularly community-acquired pneumonia, and neoplasms. Imaging examinations were conducted in 90.9% of patients, with modalities such as B-ultrasound (4.5%), X-ray (9.1%), CT scan (77.3%), MRI (9.1%), and FDG PET (9.1%). Antibiotic treatment, primarily beta-lactam drugs like amoxicillin, was administered to all patients, with a median duration of 56 days. The median follow-up time for the 22 patients was 4.8 years; however, five patients were lost to follow-up. Among the remaining 17 patients, nine were cured after anti-infective treatment (41%), one experienced a relapse during infection treatment, and seven patients died (one from sepsis secondary to bloodstream infection, one from tumor progression, and the cause of death for the remaining five patients was unknown).

## Discussion

Our study summarized the clinical characteristics of 22 elderly patients with actinomycete infections who were treated at Peking Union Medical College Hospital from 2014 to 2024. Common risk factors for actinomycete infection in the elderly included long-term smoking and drinking history, treatment with glucocorticoids and/or immunosuppressants, type 2 diabetes, and malignant tumors. Local risk factors comprised poor oral hygiene, surgery, local trauma, and IUD insertion. Pulmonary infection was the most common site of actinomycete infection in our study, accounting for 77.3% of the total cases, followed by abdominal and pelvic infections, orocervicofacial infections, and soft tissue infections. Xu et al. [17] conducted a retrospective analysis of 31 cases of actinomycete infection admitted to a tertiary hospital in southern China over 20 consecutive years. Their findings showed that actinomycete infections were predominantly diagnosed in the orocervicofacial (n=14), cardiothoracic (n=11), abdominal (n=5), and soft tissue (n=1) regions.

Although all elderly patients with pulmonary actinomycosis presented respiratory symptoms, none of the 17 elderly patients with this condition were initially suspected of having it. Most patients are initially diagnosed with community-acquired pneumonia, lung space-occupying lesions, or lung abscesses. There are also instances where interstitial pulmonary lesions due to asbestosis are suspected initially. This highlights the lack of specificity in clinical and chest imaging manifestations of pulmonary actinomycosis, particularly when lung space-occupying lesions are observed on chest CT, leading to potential misdiagnosis as lung cancer or metastasis. For elderly patients with common or local risk factors, when respiratory symptoms and lung space-occupying lesions are present on chest CT, pulmonary actinomycosis should be considered in the differential diagnosis. Confirmation of diagnosis should involve further examinations such as lesion biopsy under CT guidance or bronchoalveolar lavage fluid culture under bronchoscopy. Additionally, our study revealed that pulmonary actinomycosis can coexist with tumors such as lung squamous cell carcinoma and gastric adenocarcinoma.

In terms of soft tissue infection caused by actinomycetes, case 21 presents a noteworthy case. The patient developed necrotizing fasciitis of the right foot following a pedicure, leading to surgical debridement. Subsequent pathogenic cultures of the debrided tissue confirmed an actinomycetes infection. It is postulated that the foot infection may have been due to inadequate sterilization of pedicure equipment or contamination. Moving on to abdominal and pelvic infections, case 19 was diagnosed with infectious aortitis caused by actinomycetes infecting an artificial abdominal aortic stent, an exceedingly rare occurrence. Actinomycetes exhibit a remarkable ability to cause infections in unexpected locations, highlighting the gaps in our understanding of these pathogens.

In this study of 22 cases of actinomycete infection in elderly patients, all individuals were treated with antibiotics, primarily beta-lactam drugs such as amoxicillin. Patients with actinomycosis typically require prolonged (6 to 12 months) high doses of penicillin G or amoxicillin. However, the duration of antimicrobial therapy could potentially be reduced to three months for patients who have undergone optimal surgical resection of infected tissues. Specific preventive measures, such as reducing alcohol abuse, maintaining good dental hygiene, and changing the IUD every five years, may help limit the occurrence of actinomycosis [1]. It is crucial for patients with actinomycosis to adhere to the prescribed treatment regimen, which often involves long-term antibiotic therapy, to effectively eradicate the infection. In cases where surgical intervention is necessary, such as the removal of infected tissues, the duration of antimicrobial therapy may be shortened. This highlights the importance of a multidisciplinary approach to managing actinomycosis to ensure optimal outcomes for patients. In addition to treatment, preventive measures play a key role in reducing the risk of actinomycosis. By addressing factors such as alcohol abuse, dental hygiene, and the use of intrauterine devices, individuals can potentially minimize the likelihood of developing this condition. Education and awareness of these preventive measures are essential in promoting overall health and well-being among at-risk populations.

During the follow-up period, seven patients died, with the cause of death remaining unknown for five of these individuals. Case 19 details a 67-year-old male who underwent abdominal aortic stent implantation due to an abdominal aortic aneurysm. The patient also demonstrated poor oral hygiene, a history of recurrent gingivitis, and a prolonged history of heavy smoking, with a smoking index of 800. He was admitted to the hospital with a fever. Enhanced CT scans of the abdomen and pelvis revealed inflammatory changes in the tissue surrounding the abdominal aorta. Two blood cultures confirmed the presence of actinomycetes. Ultimately, the patient was diagnosed with infective aortitis resulting from an actinomycetes infection of the artificial stent. Despite receiving aggressive and adequate anti-infective treatment, he succumbed to sepsis caused by an actinomycete bloodstream infection. The duration from onset to death was 187 days. Case 15 describes a 67-year-old male diagnosed with actinomycosis through bronchoalveolar lavage fluid culture who was subsequently identified as having lung squamous cell carcinoma via bronchoscopic mucosal biopsy. Following aggressive anti-infective treatment, the patient's lung actinomycetosis infection was effectively resolved. He then underwent four cycles of chemotherapy, which combined cisplatin and gemcitabine. During this chemotherapy regimen, the patient experienced significant side effects, including vomiting, rapid weight loss, and a marked decline in physical function. Consequently, chemotherapy was discontinued after four cycles, and he ultimately succumbed to the progression of lung squamous cell carcinoma. The duration from onset to death was five months.

Although we provided a detailed summary of the clinical characteristics of 22 cases of actinomycete infection in elderly individuals, our study is limited in several ways. Firstly, all cases were sourced from a single center, Peking Union Medical College Hospital, which may limit the generalizability of our findings. We advocate for future multi-center, large-sample clinical studies to offer a more comprehensive understanding of actinomycete infections in the elderly population. Secondly, only one out of the 22 patients underwent a comprehensive geriatric assessment in our study. As a result, we were unable to analyze the physical functional status and nutritional status of the elderly participants, which hindered our ability to fully grasp their characteristics. It is imperative to explore the impact of functional and nutritional status on the prognosis and outcome of actinomycete infections in elderly individuals in future studies.

## Conclusions

Actinomycete infections are infrequent in elderly individuals, with clinical features and imaging findings lacking specificity. Pulmonary actinomycosis is the most prevalent form of actinomycetal infection in this age group. In elderly patients with common or local risk factors, the presence of respiratory symptoms and lung space-occupying lesions on chest CT should prompt consideration of pulmonary actinomycetes in the differential diagnosis. It is important to recognize that actinomycetes have a wide-ranging ability to cause infections beyond our current understanding. Treatment of actinomycosis typically involves high-dose penicillin. Clinicians should strive to improve their knowledge of actinomycosis to prevent delays in diagnosis and treatment.
